# I Would Speak Up to Live Up to Your Trust: The Role of Psychological Safety and Regulatory Focus

**DOI:** 10.3389/fpsyg.2019.02966

**Published:** 2020-01-23

**Authors:** Yu Song, Peng Peng, Guangtao Yu

**Affiliations:** Business School, Central University of Finance and Economics, Beijing, China

**Keywords:** feeling trusted, theory of planned behavior, psychological safety, regulatory focus, voice

## Abstract

Voicing upward refers to employee efforts to improve organizational functioning by making suggestions or expressing opinions and concerns. While extant studies have investigated how supervisors’ behaviors or attitudes influence employee voice behaviors, researchers have paid little attention to the effects of employee perceptions on voice. Based on the theory of planned behavior (TPB), we developed and tested the effects of feeling trusted by supervisors on two dimensions of voice (promotive and prohibitive), focusing on the mediation role of psychological safety and the interaction effect of psychological safety and regulatory focus on voice. Using a sample of 244 participants and three waves of longitudinal data, we investigated whether feeling trusted would lead to both promotive and prohibitive voice through psychological safety. We also extensively examined the moderation effect of regulatory focus on psychological safety and the contingency dimension of voice. We found that promotion focus strengthens the positive relationship between psychological safety and voice (both promotive and prohibitive voice), whereas prevention focus strengthens the positive relationship between psychological safety and prohibitive voice. This paper concludes with a discussion of the theoretical and practical implications of these findings.

## Introduction

Increasingly recognizing its benefits, organizations are currently devoting more and more attention to employees’ voice. Voice is an important means by which employees contribute to organizations, either by expressing constructive ideas, making suggestions, highlighting concerns, or conveying information about problems related to work issues (Liang et al., 2012; Morrison, 2014; Chamberlin et al., 2017). Prior studies have shown that voice behavior could help organizations improve performance (Morrison, 2011; Grant, 2013; Lin and Johnson, 2015), adapt to environments (Schwarz, 2003; Liang et al., 2012), deal with problems (Detert and Burris, 2007), and benefit work teams (Van Dyne and LePine, 1998; Nemeth et al., 2001). Over the years, scholars have attempted to understand the nature of voice (Van Dyne and LePine, 1998) and its boundary conditions (Avery et al., 2011), as well as the individual, contextual, and motivational antecedents that facilitate or inhibit it (Liang et al., 2012).

However, despite these voice-related research achievements, we believe current voice research needs improvement. First, while early studies devoted a great deal of attention to identifying voice antecedents independently and uniquely, recent studies have sought to identify the latent psychological mechanisms of these relationships (Morrison, 2014; Lam et al., 2018; Koopmann et al., 2019; Engemann and Scott, 2020). One plausible explanation for this is that early studies tended to identify the effect of various antecedent factors differentially and uniquely, ignoring the fact that individual, contextual, and motivational factors may impact voice synergistically (Dyne et al., 2003; Engemann and Scott, 2020). Since few studies have set out to investigate the interaction effect of the antecedents to voice, better understanding the psychological mechanisms underlying voice will require further research. With respect to individual differences, past studies have proposed that individuals’ perceived behavior control and attitude can play an important role in the casual relationship with voice; however, studies in this domain have so far lacked sufficient empirical support (e.g., Lin and Johnson, 2015; Koopmann et al., 2019). Moreover, while recent investigations into the antecedents of voice have focused on individuals’ regulatory focus as an important attitude factor that facilitates voice (e.g., Liang et al., 2012; Liu et al., 2017), unfortunately researchers have not yet examined the interaction effects of attitude and psychological factors.

Second, although previous research has framed employee voice as speaking up with constructive suggestions as well as expressing concerns regarding already existing or impending risks for the organizations (Dyne et al., 2003), the empirical research in this domain remains insufficient. In addition, scholars have recognized voice behavior as dichotomous – promotive voice and prohibitive voice are significant in different ways for organizations (e.g., Liang et al., 2012; Huang et al., 2018) – but previous research has focused heavily on promotive voice; consequently, prohibitive voice, especially its underlying psychological mechanisms and antecedents, remains underexamined. Thus, scholars still need to attempt to understand the psychological mechanisms of promotive and prohibitive voice simultaneously.

Finally, in terms of the psychological factors of voice, trust is an important but overlooked antecedent of employee voice behaviors. Some studies have revealed that trust in organizations might affect the voice behavior of employees (Duan and Tian, 2011; Hollensbe et al., 2014; Alison et al., 2015). Meanwhile, although feeling trusted has been emphasized, few studies have investigated the relationship between feeling trusted and voice behavior. Feeling trusted refers to the perception that another party (generally an individual’s direct supervisor) is willing to accept vulnerability as a result of one’s action (Baer et al., 2015). Studies have shown that the trust of leaders could influence employees’ voice behavior (Gao et al., 2011; Holley et al., 2019); in addition, scholars have asserted that feeling trusted can be exhausted for employees, thus decreasing their job performance (Baer et al., 2015). Undoubtedly, a gap in the research regarding the psychological processes that foster feelings of trust and employee voice remains to be filled.

**FIGURE 1:**
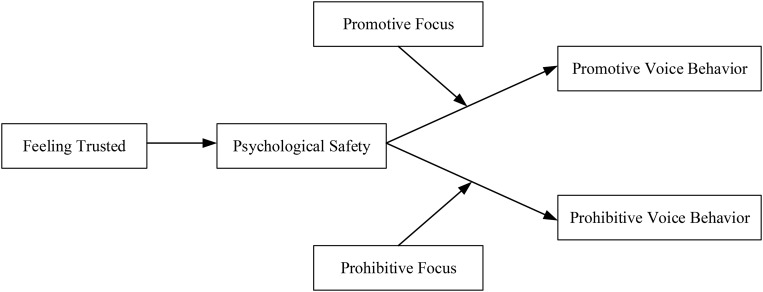
The hypothesized conceptual model.

Based on the discussion above, we designed this study to elucidate the psychological mechanisms of employee voice based on the theory of planned behavior (TPB) (Ajzen, 1991, 2005, 2012). We explored how and when feeling trusted may lead to promotive and prohibitive voice. The TPB postulates that individuals’ perceived control behavior belief influence behavior through perceived control behavior. We hypothesized that employees who feel highly trusted (i.e., perceived behavior control belief) will subjectively evaluate relationships with their leaders as positive, which will generate the feelings of psychological safety (i.e., perceived behavior control) necessary to engage in voice behavior. We further postulated that individual regulatory focus would moderate these effects. Scholars have suggested that the differentiating effects of psychological safety depend on individual attitudes (e.g., regulatory focus) that affect the degree of attention an employee devotes to enhancing his or her behavior (Liu et al., 2017; Koopmann et al., 2019). Promotion focus is concerned with accomplishments, hopes, and aspirations, whereas prevention focus is concerned with safety, responsibilities, and obligations (Higgins, 1998). We predicted that employees with high promotion focus would concentrate more on the benefits of organizations and engage in higher levels of promotive voice. Likewise, we expected that employees with high prevention focus would tend to emphasize the non-loss of organizations and engage in prohibitive voice. We conducted a three-wave study to test our theoretical model. Figure 1 depicts our theoretical framework.

This study makes several theoretical contributions to this field. First, we shed light on the relationship between feeling trusted and voice behavior. Our research suggests that employees’ effective instruments of overcoming restraint and hesitation should be the intrinsic motivators, that is, feeling trusted. Second, we identified employee levels of promotion or prevention focus as key contingencies affecting the proposed effects of feeling trusted through psychological safety. Finally, we extended and tested the TPB theory by examining the relationship between perceived behavior control belief (i.e., feeling trusted), perceived behavior control (i.e., psychological safety), attitude (i.e., regulatory focus), and behavior (i.e., voice), particularly the interaction between perceived behavior control and attitude.

## Theory and Hypothesis

### A Planned Behavior Perspective on Voice

The TPB theory is adaptive, pervasive, and vigorous in predicting individual behaviors, supported by extensive empirical research (Ajzen, 1991, 2012). According to the TPB, a given behavior is mostly determined by individual intentions, which stem from attitude, subjective norms, and perceived behavior control. Individuals’ attitudes toward a behavior reflect their perceptions of that behavior. If an individual has a favorable judgment of a behavior, the potential that he or she will enact it will be higher. Subjective norms refer to individual perceptions of social pressure to engage in a given behavior; individual normative beliefs influence subjective norms. Perceived behavior control, a non-motivational factor, which plays a pivotal role in the TPB, refers to individual perceptions of the possibility of performing a given behavior (e.g., self-efficacy). Studies have purported that attitudes, subjective norms, and perceived behavior control influence intentions uniquely, jointly, and synergistically, and that they influence behavior via the intention channel (e.g., Liang et al., 2012; Ajzen and Sheikh, 2013; Nuttavuthisit and Thøgersen, 2017). Above all, it is worth noting that on the basis of the TPB, intention and perceived behavior control can be used to predict a given behavior directly, jointly, and interactively (Ajzen, 1991, 2012).

Voice behavior is an important factor in organizational citizenship behavior (OCB), but unlike other OCBs, voice is unique and challenging (Wang et al., 2016; Chamberlin et al., 2017). Voice refers to employees speaking up to contribute to work and a recent study identified it as a dichotomous concept consisting of promotive voice (e.g., suggestion of innovative ideas) and prohibitive voice (e.g., alerts of potential risk) (Liang et al., 2012; Lin and Johnson, 2015). Employees can help implement organizational objectives via their voice behavior (e.g., Detert and Burris, 2007; Morrison, 2011; Grant, 2013). However, the differentiated consequences of employee voice make it more like a double-edged sword and, both conceptually and in terms of its consequences, it appears much more like a planned behavior (Grant, 2013; Lam et al., 2018). On the one hand, voicing ideas benefits speakers; numerous studies have identified the positive results – including visibility, favorable performance appraisal, and promotion opportunities – of voice behavior for employees (e.g., Morrison, 2011, 2014; Grant, 2013). On the other hand, even though voice behavior is important for improving and managing organizations, for employees, it can still prove risky (Morrison, 2014; Engemann and Scott, 2020). Because voice behavior tends to involve changes or challenges to the *status quo*, it can introduce risks of misunderstanding and undesired social consequences (Tangirala et al., 2013). Indeed, voice behavior intended to benefit one’s organization may easily be misinterpreted by colleagues and supervisors as “bossiness” (Tepper et al., 2004). Therefore, from the employees’ point of view, voicing is a complicated behavior – potentially beneficial or dangerous – that depends on multiple factors in interpersonal contexts. Thus, voice behavior can be defined as a sensible and planned behavior.

Generally, researchers have defined trust in an individual as the extent to which other people are willing to take risks and expose their vulnerabilities on account of said individual (Mayer et al., 1995; Rousseau et al., 1998). Trusting and feeling trusted are two sides of the same coin; feeling trusted involves the expectation that another party will tolerate one’s risky behavior (Lau et al., 2014; Baer et al., 2015). Employees who feel trusted by their leaders feel positive expectations coming from their supervisors’ intentions or behaviors (Brower et al., 2009; Baer et al., 2015).

Feeling trusted is likely to cause employees to engage in voice behavior. The TPB regards feeling trusted as an individual’s belief that another party would be willing to accept vulnerability as a result of his or her risk-taking actions (McKnight et al., 1998; Lau et al., 2014). As pointed out above, voice behavior always challenges the current *status quo* and carries the risk of producing undesired outcomes (Morrison, 2014; Nechanska et al., 2020), including perceived bossiness (Tepper et al., 2004), penalties, or punishments (e.g., Detert and Burris, 2007; Grant, 2013). Therefore, voice upward intentions among individuals who regard their relationships with their supervisors as untrusting would be considered risk-taking behavior, and this perception would serve as a decisive obstacle to the enactment of voice. Indeed, the fact that voice behavior in such circumstances involves risk-taking or personal danger may lead individuals to resist speaking up or to remain silent (Morrison, 2014). By contrast, individuals who perceive their relationships with their supervisors as supportive tend to not regard voicing upward as risk-taking and this perception enables them to engage more frequently in voice behavior. Moreover, studies have found that employees who are trusted by their supervisors are more prone to exhibit more citizenship behavior (Lau et al., 2014; Kim et al., 2018). Taking these factors into consideration, we proposed Hypothesis 1 as follows:

*Hypothesis 1:* Feeling trusted is positively related to (a) promotive voice behavior and (b) prohibitive voice behavior.

### The Mediating Role of Psychological Safety

To effectively explain the ways feeling trusted may influence voice, we imported an important antecedent of voice – psychological safety. Psychological safety refers to an individual’s perception of the relative supportiveness of a given climate (Edmondson, 1999). Based on this definition, psychological safety essentially refers to volitional control – that is, perceived behavior control: a measure of the degree to which employees perceive their work climates as safe for engaging in certain risky behaviors. Psychological safety is important for fostering teamwork and serves as a key factor in preventing potential problems; in psychologically safe climates, employees can devote their attention to the productive and constructive discussions that enable them to avoid problems and accomplish shared goals (Edmondson, 2004). We predicted that psychological safety would mediate the relationship between feeling trusted and voice.

Feeling trusted reflects the extent to which employees perceive their supervisors as willing to accept vulnerability as a result of their actions (McKnight et al., 1998); it tends to enhance the degree to which individuals perceive given contexts as safe (Lau et al., 2014). According to the TBP, feeling trusted is a behavior-related belief that could lead employees to subjectively evaluate their relationships with their leaders, thereby causing them to perceive their work environments as safe places for them to speak up (Ajzen, 1991, 2012; Schaubroeck et al., 2011).

In addition, because employees usually recognize their immediate supervisors as the agents or representatives of their organizations, feeling trusted encourages employees to pay more attention to their relationships with their supervisors as reflections of how their organizations treat them (Cruz et al., 2010; Fulmer and Ostroff, 2017). In a word, feeling trusted serves as important factor contributing to employees’ perceptions of working contexts or climates. In terms of the relationship between feeling trusted and psychological safety, previous studies have shown that trust is a key antecedent of psychological safety (e.g., Kahn, 1990; Schaubroeck et al., 2011). The trust built in teams can reduce supervision and jealousy among team members, thereby enhancing cohesion and psychological safety (Schaubroeck et al., 2011). Feeling trusted can enhance individual feelings of psychological safety (Kahn, 1990). Thus, we proposed Hypothesis 2 as follows:

*Hypothesis 2:* Feeling trusted is positively related to psychological safety.

Prior studies have examined the positive relationship between psychological safety and voice (e.g., Detert and Burris, 2007; Liang et al., 2012; Tangirala et al., 2013; Liu et al., 2017). Scholars have recognized psychological safety as an important antecedent of voice behavior. Employee psychological safety stems from their perceptions that their relationships with their leaders are positive and stable and that they can behave in ways that challenge the *status quo*, even if doing so may threaten others in their organizations in the short term.

In the TPB framework (Ajzen, 2012; Hilverda et al., 2018), psychological safety, as perceived behavior control, refers to employee perceptions of the ease or difficulty of performing behaviors of interest. Moreover, psychological safety is assumed to reflect past experience as well as anticipated impediments and obstacles, and it can predict risk-taking behavior. Specifically, in keeping with the notion that employees may choose to speak up or remain silent based on perceived benefits or costs, psychological safety refers to the perception that engaging in risky behaviors like speaking up may not result in unsatisfactory outcomes and thus serves as a key factor contributing to voice (e.g., Edmondson, 1999; Detert and Burris, 2007; Wang et al., 2018). In addition, while recognizing the potential losses of voicing upward could silence employees (Van Dyne et al., 2003), recognizing that the potential benefits outweigh the dangers would eventually lead employees to voice upward.

Contributing to the psychological mechanism of voice, we posited that psychological safety has a mediating effect on the positive relationship between feeling trusted and voice. As pointed out previously, feeling trusted refers to employee perceptions that their supervisors support their behaviors (Baer et al., 2015), while psychological safety refers to employee perceptions that, in their work climates, they are free to express themselves; thus, both concepts have in common the willingness to accept specific behaviors (Edmondson, 2004), for instance, voice. Moreover, because supervisors serve as representatives of organizations, employees who feel trusted by supervisors may perceive the context as supportive, leading them to engage in voice behavior (Holley et al., 2019). In addition, because voice often challenges the current *status quo* and voice upward may come with organizational rewards or penalties, leader behaviors tend to serve as implicit or explicit cues for employees regarding whether they should speak up for the organization or remain silent (Liu et al., 2017; Deng et al., 2019).

Thus, based on the TPB (Ajzen, 2015), when supervisors strive to establish trusting relationships with subordinates, employees tend to perceive organizational contexts as safer and more supportive; in such circumstances, employees are more likely to feel free to speak up without fearing the potential negative impacts of doing so. Therefore, the perception of trust can enhance employee perceptions of organizational context safety, leading them to engage in voice behavior. Prior research has examined psychological safety as a mediation bridge connecting leader behaviors and employee voice (e.g., Liu et al., 2017). Thus, we put forward Hypothesis 3 as follows:

*Hypothesis 3a*: Psychological safety mediates the positive relationship between feeling trusted and promotive voice behavior.
*Hypothesis 3b:* Psychological safety mediates the positive relationship between feeling trusted and prohibitive voice behavior.

### Moderating Effects of Regulatory Focus

Regulatory focus refers to the orientation individuals use to guide their behavior (Higgins, 1997). According to the regulatory focus theory, individuals use two kinds of orientation to regulate their behaviors: promotion focus and prevention focus. Promotion focus involves establishing ideal goals that motivate people to pursue their desired states (Johnson et al., 2017); it generally entails innovation and initiative in achieving goals (Gamache et al., 2015), and is expressed in emotional tones that activate positive feelings (e.g., excitement) as opposed to negative ones (e.g., dejection) (Higgins et al., 1997; Brockner and Higgins, 2001; Manczak et al., 2014). Prevention focus refers to the propensity to concentrate on avoiding negative outcomes such as risks, responsibilities, and obligations, and minimizing losses and financial costs (Liang et al., 2012). Prevention focus entails attention to avoiding undesirable problems and losses, and an avoidance-based awareness that identifies undetected problems. It is connected with emotional tones that tend to activate negative feelings (e.g., anxiety) as opposed to positive ones (e.g., quiescence) (Higgins, 1997).

Based on the TPB, voice behavior is a planned behavior involving an array of antecedents that act uniquely, differentially, and interactively (Liang et al., 2012). As pointed out above, three factors can influence individuals’ intentions to engage in planned behaviors: first, individuals must have positive attitudes toward the behavior, which usually combines with positive evaluations (*positive attitude*); second, individuals’ intentions to enact given behaviors must align with the restrictions imposed by normative pressure-based behavioral expectations (*subjective norms*); and third, individuals must perceive that they have overall control over the specific behaviors, which means they must be confident that they can control the behaviors (*perceived behavioral control*). Moreover, in line with the TPB, an interaction effect between attitude and perceived behavior control exists in predicting given behaviors (Ajzen, 1991, 2012).

Psychological safety contributes to perceived behavioral control of voice. According to the TPB (Ajzen, 1991, 2012), psychological safety is a key component of perceived behavioral control, which refers to the extent to the belief of individual toward to performing a given behavior, such as voice. Psychological safety concerns beliefs regarding risky consequences in working contexts, especially interpersonal risks stemming from given behaviors. Psychological safety is essential for individuals to feel capable of changing their behaviors (Edmondson, 1999, 2004). As discussed above, voice is a form of personal initiative that may result in positive consequences (such as promotion opportunities at work) or negative consequences (such as being recognized as a challenge to the *status quo*). To clarify perceived behavioral control of voice, employees often apply important channels as sourcing from their immediate interpersonal networks (i.e., supervisors and coworkers) to help them to determine the extent to which they will be viewed favorably if they express themselves at work (Liang et al., 2012; Jonczyk et al., 2016; Singh et al., 2018). In other words, it is worth noting that being acknowledged of if it is favored to enact voice, voicing without fearing of the negative consequences is regular within organizational context or not. Thus, based on this conceptual foundation, psychological safety appears to serve as an important ingredient in perceived behavioral control, determining individuals’ capability to engage in voice behavior. Following this reasoning, researchers have regarded psychological safety as contributing to voice because perceptions of psychological safety increase the ease and reduce the felt risk of expressing new ideas (Kahn, 1990; Ashford et al., 1998; Edmondson, 1999; Liang et al., 2012), which empowers individuals’ capabilities to voice upward.

While promotive focus contributes to positive attitudes regarding promotive voice, prevention focus contributes to positive attitudes regarding prohibitive voice. According to the TPB, attitude is an important antecedent of intention, and behavioral beliefs, evaluations, and behavioral inclinations are key components of attitude with no priorities (Ajzen, 1991, 2012; Ajzen and Dasgupta, 2015). Regulatory focus refers to individuals’ orientation or inclination to regulate their behaviors: promotion focus drives individuals to pursue positive outcomes; prohibitive focus drives individuals to prevent negative outcomes (Higgins, 2012). We posited that regulatory focus influences the three key components of attitude. First, the basic principle of regulatory focus theory is that people approach pleasure and avoid pain, which underlies the expectancy-value model of motivation (Higgins, 1997), while promotion focus involves the pursuit of positive outcomes and prevention focus centers on the avoidance of negative consequences, both connect to corresponding outcomes. Second, the value of each outcome differs according to the regulatory focus of individuals: individuals with high promotion focus value the attainment of positive outcomes, while individuals with high prevention focus value the avoidance of negative outcomes. Finally, regulatory focus evidently induces different strategic inclinations, which may affect attitudes toward given behaviors. Specifically, promotion focus contains an awareness of the presence or absence of positive outcomes; thus, promotion self-regulation presumably involves an inclination to approach promotion focus-based aspirations and accomplishments. In contrast, prevention focus involves sensitivity to the presence or absence of negative outcomes; therefore, prevention self-regulation presumably involves an inclination toward minimizing responsibilities and maximizing safety (Higgins et al., 1994, 1997). Moreover, regulatory focus determines individuals’ initiative to enact specific behaviors (Gamache et al., 2015). Empirical studies have examined the relationship between each kind of focus and corresponding voice behaviors: promotion focus is related to promotive voice behaviors, whereas prevention focus is related to prohibitive voice behaviors (Lin and Johnson, 2015; Chamberlin et al., 2017; Koopmann et al., 2019). Based on the notion that promotion and prevention focus are orthogonal (Lanaj et al., 2014) and have links to differentiated behaviors, we posited that regulatory focus would be much more as positive attitudes over voice, respectively.

However, studies regarding voice behaviors have highlighted the importance of acknowledging the person-based antecedents that shape voice behavior (Lin and Johnson, 2015). Although Liang et al. (2012) found that organization-based perceptions such as psychological safety could impact promotive and prohibitive voice, identifying person-based antecedents is more important that the person-based antecedents like propensity determines the individuals’ intention to a particular behavior. Thus, while organization-based factors might impact employee willingness to engage in voice behaviors, person-based antecedents should be considered. Psychological safety determines whether individuals perceive their contexts as supportive or risky for engaging in voice behaviors; meanwhile, regulatory focus drives individuals to pay attention to specific kinds of voice behaviors, meaning individuals hold particular intentions or motivations toward given behaviors. In terms of the Planned Behavior Theory, the interaction effect of attitude and perceived behavior control could synergistically influence particular behaviors (Ajzen, 1991, 2012). Thus, we posited that psychological safety and regulatory focus might impact voice behavior differently, uniquely, and interactively. Promotion focus individuals working in conditions characterized by high psychological safety will most likely engage in promotive voice. On the other hand, prevention focus individuals working in conditions characterized by high psychological safety will most likely engage in prohibitive voice. Numerous empirical studies have found that voice behavior is a result of an interaction process involving various factors. Individual voice behavior results from the interaction of personal traits, leadership, and organizational situation factors (Tangirala et al., 2013; Duan et al., 2017). Scholars have pointed out that voice behavior is the interactional outcome of personal factors and situational factors (LePine and Van Dyne, 1998). Thus, based on both the Planned Behavior Theory and the Regulatory Focus Theory as well as the literature review, we proposed Hypothesis 4 as follows:

*Hypothesis 4:* Regulatory focus moderates the relationship between psychological safety and voice behavior. Specifically, (a) promotion focus moderates the relationship between psychological safety and promotive voice – the stronger an employee’s promotion focus, the more positive this relationship, and (b) prevention focus moderates the relationship between psychological safety and prohibitive voice – the stronger an employee’s prevention focus, the more positive this relationship.

## Methods

### Ethics Statement

This study was carried out in accordance with the recommendations of ethical guidelines of the Ethical Review Board of Central University of Finance and Economics. The protocol was approved by the Ethical Review Board of Central University of Finance and Economics. All subjects gave written informed consent in accordance with the Declaration of Helsinki.

### Sample and Procedure

To test our hypotheses, we administered questionnaires in Northern China. The sample participants primarily worked on the construction, finance, and manufacturing, such as junior engineers, auditors, and skilled workers, and the like. They had frequent interactions with their supervisors, who were in charge of assigning tasks and monitoring. For two reasons, this is an ideal setting for investigating trust and voice. One is that China, as a collectivistic culture, emphasizes trust and safety in its culture (House et al., 2004). The other is, whether junior engineers, auditors, or skilled works, they are all knowledge workers, who both need some autonomy, which comes from supervisors’ trust, and are expected to point out potential issues to improve effectiveness (Janz et al., 1997). Additionally, recent research provides evidence that the Chinese context is appropriate for researching voice (e.g., Xin et al., 2015; Liu et al., 2017; Huang et al., 2018). Thus, given the nature of the context, the assessment of employee voice is of particular interest.

We conducted three waves to collect data. Following Singer and Willett’s (2003) recommendation, we spaced the waves of data collection in such a way as to capture the meaning of measured variables during these determined periods of time. Recent research provides evidence that the 2 weeks allows for forming and developing perceptions of our variable of interest (e.g., Fulmer and Ostroff, 2017; Liu et al., 2017; Kim et al., 2018). In addition, based on our discussions with senior management, 2-week lag between every wave of the survey could synchronize with the weekly or 2-weekly employees’ regular meetings. Thus, each wave of our survey was separated by 2 weeks. We asked participants to finish each questionnaire within 20 min and paid them 5 RMB yuan per wave for participation. We informed the participants that their identities would be kept anonymous and encouraged them to respond to the questionnaires truthfully. In phase 1, we sent questionnaires to 427 participants and asked them to report their demographics and rate how trusted they felt. Two weeks later, we asked the 294 participants who responded in phase 1 to rate their psychological safety. After another 2 weeks, we asked the 261 participants who responded in the first two rounds to report their regulatory focus and voice behavior.

Our final sample consisted of 244 valid responses, with an overall response rate of 57.14%. Because we collected data in November and December which are two of the busiest months for most Chinese companies, 183 participants drop out in the second or third wave of data collection for reasons such as fatigue or because they were too busy at work. We conducted a multivariate analysis of variance to examine whether participants’ response versus non-response created any detectable differences in our sample (Lance et al., 2000). Results showed that participants in the initial randomly selected sample and in the final sample used for model testing did not differ significantly with regard to age, *t*(425) = −0.10, *p* = 0.92, title, *t*(425) = 1.55, *p* = 0.12, education, *t*(425) = 1.52, *p* = 0.13, organizational tenure, *t*(425) = −1.08, *p* = 0.28, or gender, χ^2^(1) = 2.47, *p* = 0.12. Participants’ average age was 33.10 years (range = 22–60 years); average tenure at the organization was 4.16 years (range = 1–36 years); 27.87% were female; 89.75% had college degrees. Participants consist of 134 employees (54.92%), 55 supervisors (22.54%), 49 middle managers (20.08%), and six top managers (2.46%).

### Measures

Survey items were back-translated following the procedure developed in Brislin (1986). We used a response format of 1 = *strongly disagree* to 5 = *strongly agree*.

#### Feeling Trusted

We used Lau et al.’s (2014) 10-item scale to measure the two dimensions (reliance and disclosure) of feeling trusted. Sample items include: “My supervisor relies on my task-related skills and abilities” and “My supervisor discusses work-related problems or difficulties that could potentially be used to disadvantage him/her.” The Cronbach’s alpha coefficient was 0.88.

#### Psychological Safety

We used Liang et al.’s (2012) three-item scale to measure psychological safety. Sample items include: “In my work team, I can express my true feelings regarding my job,” “In my work team, I can freely express my thoughts,” and “In my work team, expressing your true feelings is welcomed.” The Cronbach’s alpha coefficient was 0.83.

#### Regulatory Focus

We used Higgins et al.’s (2001) 11-item scale to measure regulatory focus. We assessed promotion focus with six items. Sample items include: “Compared to most people, are you typically unable to get what you want out of life?,” “Do you often do well at different things that you try?,” and “I feel like I have made progress toward being successful in my life.” The Cronbach’s alpha coefficient was 0.85. We assessed prevention focus with five items. Sample items include: “Growing up, would you ever ‘cross the line’ by doing things that your parents would not tolerate?,” “How often did you obey rules and regulations that were established by your parents?,” and “Growing up, did you ever act in ways that your parents thought were objectionable?.” The Cronbach’s alpha coefficient was 0.86.

#### Voice Behavior

We measured voice behavior using Liang et al.’s (2012) 10-item scale. We assessed promotive voice behavior with five items. Sample items include: “Proactively develop and make suggestions for issues that may influence the team,” “Proactively suggest new projects that are beneficial to the work team,” and “Make suggestions to improve the team’s working procedures.” The Cronbach’s alpha coefficient was 0.90. We assessed prohibitive voice behavior with five items. Sample items include: “Speak up honestly about problems that might cause serious loss to the work team, even when/though dissenting opinions exist,” “Dare to voice opinions on things that might affect efficiency in the work team, even if that would embarrass others,” and “Dare to point out problems when they appear in the team, even if that would hamper relationships with other colleagues.” The Cronbach’s alpha coefficient was 0.86.

#### Control Variables

We included age, gender, education level, organizational tenure, and position in one’s organization as control variables because of their potential impact on voice. Prior research examining how gender influences employees’ voice behavior has suggested that females may be less likely to speak up than males (Tangirala et al., 2013; Duan et al., 2017). In addition, because employees with longer organizational tenures are more confident about their own standing as organizational members and more familiar with their supervisors’ leadership styles, they are more confident in engaging in voice behaviors (Takeuchi et al., 2012). Similarly, employees in higher organizational positions may feel more obligated to speak up and engage in more upward voice (Morrison, 2014). We measured position in the organization using four categories: employees, supervisor, middle managers, and top managers. Likewise, we measured education level using four categories: high school or below, junior college, undergraduate, and graduate or above.

## Results

### Descriptive Statistics

Table 1 shows the descriptive statistics and zero-order correlations for our variables. The coefficient alphas appear in parentheses on the diagonal. An examination of the zero-order correlations provided initial support for our hypotheses. Feeling trusted was positively related to psychological safety, *r* = 0.26, *p* < 0.001, and promotive voice, *r* = 0.31, *p* < 0.001, as well as prohibitive voice, *r* = 0.27, *p* < 0.001. Psychological safety was positively related to promotive voice, *r* = 0.34, *p* < 0.001, and prohibitive voice, *r* = 0.32, *p* < 0.001.

**TABLE 1:** Means, standard deviation, and correlations among the study variables.

Variables	*M*	*SD*	1	2	3	4	5	6	7	8	9	10	11
(1) T1 AGE	33.10	7.17	−										
(2) T1 GENDER	0.28	0.45	–0.01	−									
(3) T1 TITLE	1.70	0.87	0.33***	–0.12	−								
(4) T1 EDU	3.25	0.68	−0.21*	–0.16	–0.05	−							
(5) T1 TENURE	4.16	4.48	0.64***	0.08	–0.05	−0.23***	−						
(6) T1 FT	5.35	0.93	0.00	0.03	0.11	0.16	–0.03	(0.88)					
(7) T2 PS	3.96	0.75	–0.02	–0.01	–0.04	0.14	0.10	0.26***	(0.83)				
(8) T3 PMF	3.76	0.81	0.09	0.04	–0.06	0.04	0.08	0.16	0.19**	(0.85)			
(9) T3 PEF	3.76	0.73	0.08	0.02	–0.06	0.06	0.06	0.19**	0.18*	0.23***	(0.86)		
(10) T3 PMV	4.25	0.59	0.11	–0.02	0.07	–0.02	0.07	0.31***	0.34***	0.25***	0.27***	(0.90)	
(11) T3 PHV	3.64	0.75	0.20*	0.03	0.12	–0.12	0.17*	0.27***	0.32***	0.30***	0.30***	0.53***	(0.86)

*N* = 244. “FT” is feeling trusted, “PS” is psychological safety, “PMF” is promotion focus, “PEF” is prevention focus, “PMV” is promotive voice behavior, “PHV” is prohibitive voice behavior. T1, T2, and T3 refer to the time wave variables collected at phases 1, 2, and 3, respectively. Cronbach α in parentheses. *p < 0.05, **p < 0.01, ***p < 0.001.

### Confirmatory Factor Analyses

We conducted confirmatory factor analyses (CFAs) using Mplus 7.4, taking the clustered nature of our sample into account and using robust maximum likelihood estimation. Our initial measurement model contained six factors (i.e., feeling trusted, psychological safety, promotion focus, prevention focus, promotive voice, and prohibitive voice). As Table 2 shows, this model had a good fit with the data: χ^2^(422) = 784.50, *p* < 0.001, comparative fit index (CFI) = 0.93, Tucker–Lewis index (TLI) = 0.92, root-mean-square error of approximation (RMSEA) = 0.06, and standardized root mean square residual (SRMR) = 0.06. We then tested an alternative four-factor model. The only difference between this model and our first one was that, in this one, we combined promotion focus and prevention focus into one factor and promotive voice and prohibitive voice into one factor. The fit of this model was significantly inferior to that of our proposed model, χ^2^(456) = 1742.07, *p* < 0.001, and the overall fit indices were unacceptable (CFI = 0.72, TLI = 0.70, RMSEA = 0.11, SRMR = 0.09). Subsequently, we tested a two-factor model in which feeling trusted and psychological safety loaded onto one factor. The fit of this model was also significantly inferior to that of our proposed four-factor model, χ^2^(463) = 2845.28, *p* < 0.001, and the overall fit indices were also unacceptable (CFI = 0.49, TLI = 0.45, RMSEA = 0.15, SRMR = 0.13). A final model in which all measures loaded onto one factor also had a significantly inferior fit than our four-factor model, χ^2^(464) = 3349.54, *p* < 0.001, and the fit indices were unacceptable by any standard (CFI = 0.38, TLI = 0.33, RMSEA = 0.16, SRMR = 0.14). In sum, the CFAs indicated that our proposed six-factor model fit the data well, and the fit of this model was clearly superior to that of simpler models. This supported the validity of our specified measurement model.

**TABLE 2:** Results of confirmatory factor analysis of study variables.

Model	χ2	*df*	Δχ2	Δ*df*	CFI	TLI	RMSEA	SRMR
(1) Six-factor	784.50	442	−	−	0.93	0.92	0.06	0.06
(2) Four-factor	1742.07	456	957.57	14	0.72	0.70	0.11	0.09
(3) Two-factor	2845.28	463	2060.78	21	0.49	0.45	0.15	0.13
(4) One-factor	3349.54	464	2565.04	22	0.38	0.33	0.16	0.14

*df = degrees of freedom; Δχ2=chi-square differences; Δdf = degrees of freedom differences; CFI = comparative fit index; TLI = Tucker–Lewis index; RMSEA = root-mean-square error of approximation; SRMR = standardized root-mean-square residual. *N* = 244. Model 1 (six-factor model) includes all study variables. Model 2 (four-factor model) combines promotion focus and prevention focus as one factor and promotive voice behavior and prohibitive voice behavior as one factor, and considers the other two variables as two independent factors. Model 3 (two-factor model) combines feeling trusted and psychological safety as one factor and the other four variables as another factor. Model 4 (one-factor model) combines all six variables as one factor. Δχ^2^ and Δdf reflect differences of χ^2^ and df between the corresponding model and Model 1. All χ^2^ and Δχ^2^ are significant at *p* < 0.001 level.*

### Test of Hypotheses

We tested all hypotheses using structural equation modeling in Mplus 7.4. As summarized in Table 3, after including the controls, employees’ feeling trusted was positively related to psychological safety (β = 0.36, *p* < 0.001), supporting Hypothesis 2. In addition, the relationship between feeling trusted and promotive voice behavior was significant (β = 0.33, *p* < 0.001), and the indirect effect of feeling trusted on promotive voice through psychological safety was significant (indirect effect = 0.07, *p* < 0.005); thus, psychological safety partially mediated the relationship between feeling trusted and promotive voice, supporting Hypotheses 1a and 3a. Similarly, the relationship between feeling trusted and prohibitive voice was significant (β = 0.24, *p* < 0.005), and the indirect effect of feeling trusted on prohibitive voice through psychological safety was significant (indirect effect = 0.10, *p* < 0.005); thus, the relationship between feeling trusted and prohibitive voice was partially mediated by psychological safety, supporting Hypotheses 1b and 3b.

**TABLE 3:** Summary of hypotheses 1–3 results.

Variables	Psychological safety			Promotive voice behavior			Prohibitive voice behavior		
			
	*b*	SE *b*	β	*B*	SE *b*	β	*b*	SE *b*	β
**Controls**									
Age	–0.01	0.01	–0.13	0.01	0.01	0.11	0.01	0.01	0.13
Gender	–0.05	0.11	–0.03	–0.06	0.08	–0.04	0.00	0.10	0.00
Title	–0.01	0.06	–0.02	0.00	0.05	0.00	0.06	0.06	0.06
Education	0.11	0.08	0.10	–0.08	0.06	–0.09	–0.17	0.07	−0.15*
Tenure	0.04	0.02	0.21*	0.00	0.01	–0.03	0.01	0.01	0.03
**Independent variable**									
Feeling trusted	0.37	0.11	0.36***	0.27	0.10	0.33***	0.26	0.11	0.24**
Mediator									
Psychological safety				0.19	0.06	0.24**	0.25	0.08	0.25**
Indirect effect				0.07**	0.03		0.10**	0.04	
*R*2	0.17**	0.06		0.23***	0.06		0.23***	0.06	

*N* = 244. Gender was coded as 0 = male, 1 = female. Title was coded as 1 = employee, 2 = supervisor, 3 = middle manager, 4 = top manager. Education was coded as 1 = high school or below, 2 = junior college, 3 = undergraduate, 4 = graduate or above. Model fit statistics: χ^2^(12) = 15.28, CFI = 0.99, TLI = 0.96, RMSEA = 0.03, SRMR = 0.03. *b* = unstandardized coefficient; SE = standard error; β = standardized coefficient. *p < 0.05, **p < 0.01, ***p < 0.001.

**FIGURE 2:**
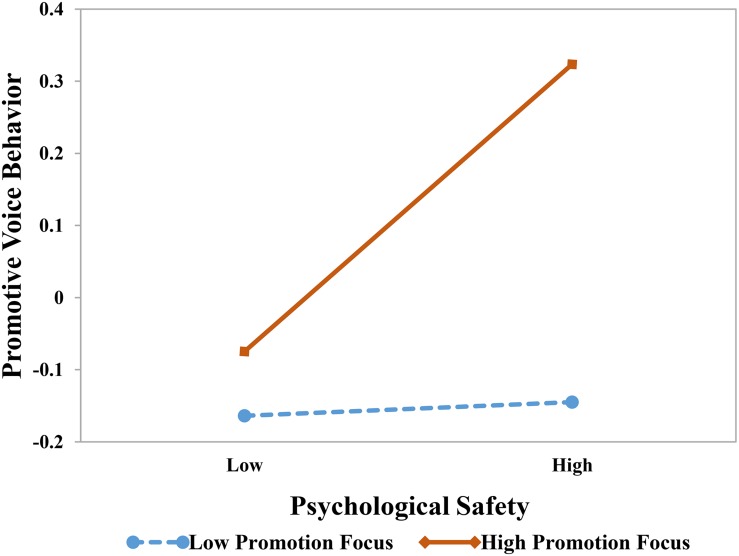
The effect of psychological safety on promotive voice behavior at high and low levels of promotion focus. The simple slope tests showed that the relationship between psychological safety and promotive voice behavior was more positive for individuals with high promotion focus (*b* = 0.32, *p* < 0.01) than for individuals with low prevention focus (*b* = −0.16, n.s).

**FIGURE 3:**
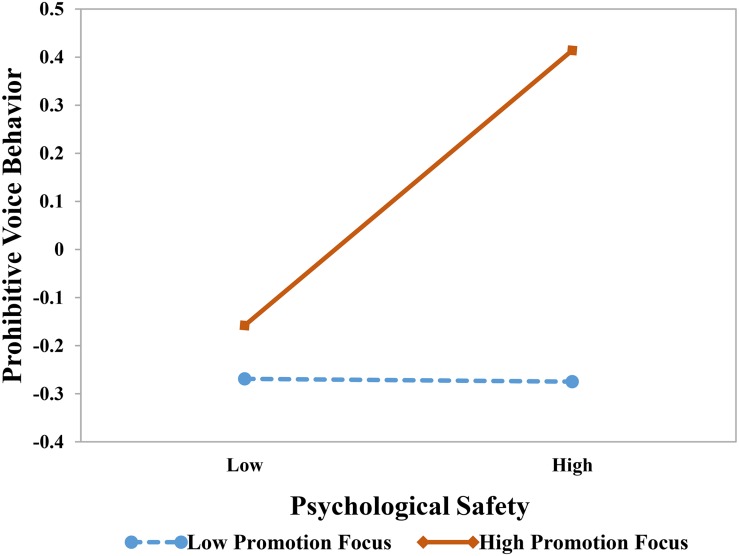
The effect of psychological safety on prohibitive voice behavior at high and low levels of promotion focus. The simple slope tests showed that the relationship between psychological safety and prohibitive voice behavior was more positive for individuals with high promotion focus (*b* = 0.41, *p* < 0.005) than for individuals with low prevention focus (*b* = −0.27, n.s).

Hypotheses 4a and 4b predicted that employees’ regulatory focus would moderate the effect of psychological safety on voice. Table 4 shows the results of these moderation effects. We found a positive interaction between psychological safety and promotion focus on promotive voice behavior (β = 0.25, *p* < 0.01). We plotted the relationships between psychological safety and promotive voice at high and low levels of promotion focus (1 *SD* above and below the mean). As Figure 2 shows, the simple slope tests indicated that the relationship between psychological safety and promotive voice was more positive with high promotion focus (*b* = 0.32, *p* < 0.01) than with low promotion focus (*b* = −0.16, n.s)., meaning the relationship between psychological safety and promotive voice is stronger in the condition of high promotion focus, supporting Hypothesis 4a. Moreover, we found that the interaction between psychological safety and promotion focus was significantly positive on prohibitive voice (β = 0.30, *p* < 0.005); Figure 3 provides a visual depiction of the simple slopes.

**TABLE 4:** Summary of Hypotheses 4 results.

Variables	Psychological safety			Promotive voice behavior			Prohibitive voice behavior		
			
	*b*	SE *b*	β	*b*	SE *b*	β	*b*	SE *b*	β
**Controls**									
Age	–0.01	0.01	–0.13	0.01	0.01	0.06	0.01	0.01	0.06
Gender	–0.05	0.11	–0.03	–0.08	0.08	–0.06	–0.04	0.09	–0.02
Title	–0.01	0.06	–0.02	0.03	0.05	0.04	0.10	0.05	0.12†
Education	0.11	0.08	0.10	–0.04	0.06	–0.05	–0.12	0.06	−0.11†
Tenure	0.04	0.02	0.21*	–0.01	0.01	–0.04	0.01	0.01	0.04
**Independent variable**									
Feeling trusted	0.36	0.11	0.35***	0.16	0.10	0.21*	0.10	0.09	0.11
Mediator									
Psychological safety				0.14	0.06	0.19*	0.19	0.06	0.19**
Moderator									
Promotion focus				0.23	0.07	0.24**	0.33	0.08	0.27***
Prevention focus				0.08	0.04	0.11	0.16	0.05	0.17**
**Interaction term**									
Psychological safety × Promotion focus				0.21	0.07	0.25*	0.32	0.08	0.30**
Psychological safety × Prevention focus				–0.07	0.06	–0.07	0.14	0.08	0.11†
*R*2	0.16**	0.06		0.24***	0.03		0.34***	0.03	

Note. *N* = 244. Gender was coded as 0 = male, 1 = female. Title was coded as 1 = employee, 2 = supervisor, 3 = middle manager, 4 = top manager. Education was coded as 1 = high school or below, 2 = junior college, 3 = undergraduate, 4 = graduate or above. The moderation and moderated mediation hypotheses were tested simultaneously. All continuous predictors were mean-centered (Edwards and Lambert, 2007). b = unstandardized coefficient; SE = standard error; β = standardized coefficient. †*p* < 0.10, *p < 0.05, **p < 0.01, ***p < 0.001.

**FIGURE 4:**
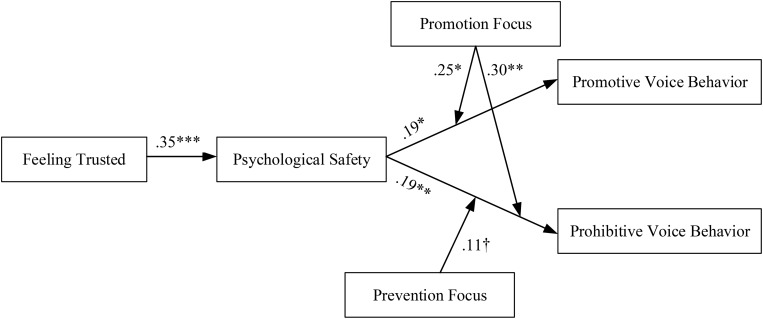
Results of structural equation modeling on voice. ^†^*p* < 0.10, *^∗^p* < 0.05, *^∗∗^p* < 0.01, *^∗∗∗^p* < 0.001.

In addition, the interaction between psychological safety and prevention focus was marginally significantly positive on prohibitive voice behavior (β = 0.11, *p* = 0.066). Similarly, the moderation effect of prevention focus on promotive voice was negative yet non-significantly (β = −0.07, n.s). Therefore, Hypothesis 4b was basically supported. Figure 4 provides a summary of the estimates of the hypothesized relationships among the variables.

## Discussion

Based on the TPB, we theoretically linked employees’ feeling trusted to both promotive voice and prohibitive voice via psychological safety. The results of our multi-wave study revealed that feeling trusted could facilitate employees’ voice behavior through the psychological safety mechanism, and employees’ regulatory focus moderated the effect of psychological safety on voice behavior. We found that employees with stronger promotion focus are more likely to resort to both promotive and prohibitive voice behavior, while employees with stronger prevention focus are inclined to engage in prohibitive voice behavior.

### Theoretical Implications

This study makes several theoretical contributions to the field. First, our research sheds light on the relationship between feeling trusted and voice behavior. We suggest that overcoming restraint and hesitation is vital in stimulating voice behaviors among subordinate employees and that intrinsic motivators, such as feeling trusted, serve as effective instruments for overcoming these obstacles. Nearly all previous studies of voice have focused on extrinsic motivators, such as leadership (Xu et al., 2015; Lam et al., 2016; Qian et al., 2018) and organizational support (Engemann and Scott, 2020). Although feeling trusted derives from supervisors’ trust in subordinates, unlike extrinsic motivators, it involves employees perceiving that their supervisors are willing to accept vulnerability and listen to their opinions. Feeling trusted may make employees feel better about themselves and more responsible for their work, giving them a sense of ownership over their jobs (Deutsch Salamon and Robinson, 2008). We found that, by building psychological safety, feeling trusted contributes in a unique way to promotive or prohibitive voice behavior. Thus, our study enriches scholarly understanding of the role feeling trusted plays in voice behavior.

Second, this study’s identification of individuals’ regulatory focus as a key contingency is another important contribution. Interestingly, we found that the ways psychological safety relates to promotive voice and prohibitive voice differ depending on individuals’ regulatory focus. Specifically, for individuals with high promotion focus, psychological safety was positively related to both promotive voice and prohibitive voice behavior. In contrast, for individuals with high prevention focus, psychological safety was only positively related to prohibitive voice behavior. Differences in the constructs of promotion focus and prevention focus might explain this finding. Promotion focus is concerned with accomplishments, hopes, and aspirations that regulate the presence and absence of positive outcomes and foster prosperity (Higgins, 1998). Whether using promotive or prohibitive voice, individuals with high promotion focus seek to maintain and improve organizations to reach ideal states. Thus, as long as positive organizational outcomes are absent, employees with high promotion focus will speak up, regardless of voice attributions. However, prevention focus is concerned with safety, responsibilities, and obligations. It regulates the presence of negative outcomes and facilitates survival (Higgins, 1998). When an environment is not safe, employees with high prevention focus can only engage in prohibitive voice behavior or remain silent.

Third, our study extended and tested the TPB theory by examining the relationship between control belief (i.e., feeling trusted), perceived behavior control (i.e., psychological safety), attitude (i.e., regulatory focus), and behavior (i.e., voice), focusing particularly on the interaction between perceived behavior control and attitude. Previous studies of the TPB have mostly focused on examining the antecedents of behaviors, neglecting the interactions between perceived behavior control and other antecedents (e.g., Hurtz and Williams, 2009; Nuttavuthisit and Thøgersen, 2017). Perceived behavior control is the most vital factor in the TPB; it refers to individuals’ perceptions of the ease or difficulty of performing the behavior of interest (Ajzen, 1991). Perceived behavior control is a unique factor that can moderate the relationship between other antecedents and behavior (Ajzen, 2012). Our empirical research establishes a link between psychological safety, regulatory focus, and voice, and demonstrates that regulatory focus can moderate the effect of psychological safety on voice.

### Practical Implications

Our study has shown that feeling trusted effectively promotes employees’ psychological safety and, thus, voice. This result can serve as advice to organizations that managers should pay attention to employees’ feelings. Although feeling trusted derives from supervisors’ trust, employees may not realize the unspoken willingness and expectations, or misinterpret the intention underlying the supervisors’ trusting actions (Lau et al., 2014). To fill up this asymmetry, managers should explicitly express trust and pay attention to actions that can directly demonstrate trust, such as knowledge sharing (Nguyen and Rose, 2009), career support (Chua et al., 2008), and empowerment (Lee et al., 2018).

Based on our findings regarding the moderating roles of employees’ promotive focus, managers should be aware of whether employees possess promotive focus. When employees with promotive focus feel strongly trusted, they will display even stronger tendencies to express their thoughts, via both promotive and prohibitive voice. Furthermore, our findings suggest that managers should more frankly display trust for employees with prevention focus because these employees engage in prohibitive voice behaviors, concentrating on escaping from and rectifying negative situations, and hearing the voices of different people could add wisdom to managers’ decisions.

### Potential Limitations and Future Directions

This study had several limitations that highlight avenues for future research. First, although our TPB-based study helps elucidate how feeling trusted affects voice via the mediating process of psychological safety and the moderating process of regulatory focus, we recognize that our research did not contain the other key antecedent (i.e., subjective norms) in the model. Subjective norms are social factors – the perceived social pressure to perform or not perform behaviors, such as psychological contracts (Bal et al., 2016). We invite future researchers to consider additional interaction effects on voice based on the TPB, and to further verify the TPB.

Second, the extent to which our findings are culturally specific warrants consideration. We found that psychological safety extends from feeling trusted and is a key mechanism in facilitating voice behavior; we also found that promotion focus amplifies the relationship between psychological safety and voice (both promotive and prohibitive voice), while prevention focus only amplifies its effects relative to prohibitive voice. The fact that our research data come from China could be significant; Chinese people are guided by the Confucian value of harmony, meaning Chinese subordinates are more likely to express hopes than worries (Huang et al., 2005). So, it is questionable whether our results could be extended to other cultures. Future research should explore whether our findings can be replicated in other cultures.

Third, our research focused on individual level, which showed that employees’ psychological safety based on feeling trusted by leaders plays a salient role in eliciting voice. A valuable extension to our research would be to examine our model on an organizational level, more specifically, whether organizational psychological safety climate can promote employees voicing freely in public, such as internal network community (e.g., ALiway Community of Alibaba, Xinsheng Community of Huawei) rather than voicing directly to their leaders. Based on Generation Z who grow with net-gen and digital natives (Turner, 2015), and are becoming the majority in workplace, they may be more likely to use online social media to express their points or comments (Cortini, 2009; Cortini and Fantinelli, 2018); therefore, future studies, especially those with a focus on Generation Z, should explore how to promote them to speak and voice freely online without fearing to be dooced.

Finally, our efforts to minimize the proportion of non-responses when we designed our study may have been insufficient. The three waves of the survey received a relatively low response rate. Low response rate may have attenuated certain observed relationships when we tested the model. To avoid this potential problem, future studies could increase response rates by using shorter surveys. In addition, obtaining stronger endorsements from management could encourage employees to complete and return the surveys.

## Conclusion

This study extends the current understanding of the impact of feeling trusted by supervisors on employees and represents an initial attempt to explore the effect of feeling trusted on voice behavior. Specifically, drawing on the extended TPB framework and identifying psychological safety as the vital linking mechanism, we connected feeling trusted to both promotive and prohibitive voice behavior. We also found that promotion focus amplifies the contributions of feeling trusted to promotive and prohibitive voice behavior. These findings provide a novel perspective on the psychological processes by which feeling trusted by supervisors shapes employees’ voice behavior. We hope that our study will spur further explorations of feeling trusted.

## Data Availability Statement

The datasets generated for this study are available on request to the corresponding author.

## Author Contributions

All authors were involved in the writing of the theoretical background and discussion sections. YS and PP collected the data, made statistical analyses, and wrote the manuscript.

## Conflict of Interest

The authors declare that the research was conducted in the absence of any commercial or financial relationships that could be construed as a potential conflict of interest.
